# Tumour immunoprophylaxis in mice using glutaraldehyde-treated syngeneic myeloma cells.

**DOI:** 10.1038/bjc.1981.81

**Published:** 1981-04

**Authors:** S. Ben-Efraim, R. Ophir, E. H. Relyveld


*Br. J. Cancer* (1981) **43**, 554

***Short Communication***

### TUMOUR IMMUNOPROPHYLAXIS IN MICE USING GLUTARALDEHYDE-TREATED SYNGENEIC MYELOMA CELLS

S. BEN-EFRAIM*, R. OPHIR* AND E. H. RELYVELD†

*From the *Department of Human Microbiology, Sackler School of Medicine, Tel-Aviv University, Tel-Aviv, Israel and the †Institut Pasteur, Annexe de Garches,* 92380 *Garches, France*

Received 25 July 1980 Accepted 16 December 1980

ATTEMPTS to induce antitumour protection with syngeneic tumour cells treated with glutaraldehyde (GA) have been reported by numerous workers. Induction of antitumour protection was reported in some cases (Csaba, 1967; Powell, 1975; Sanderson & Frost, 1974; Frost & Sanderson, 1975; Frost *et al.*, 1976; Kataoka *et al.*, 1975, 1977*a*, *b*, 1978, 1979*a*, *b*, *c*, *d*; Tomecki, 1979) whereas in other systems no protection was achieved (Kluchareva *et al.*, 1978; Price *et al.*, 1979). The use of GA was based on its property as a stabilizer of the cell surface by virtue of its activity as a protein cross-linking reagent (Richard & Knowles, 1968). It has

TABLE.—*Immunization of BALB/c mice with GA-treated MOPC*-315 *plasmacytoma cells*

| | | | Immunization procedure** | | Take | | | Mortality†† | |
|---|---|---|---|---|---|---|---|---|---|
| Pool Number | No. of Expts | No. of groups* | No. inj. | First injection (%GA) | +ve/ total | MTD† (days) | *P*††† | MTM (days) | *P* |
| I | 5 | 9 | 2 or 4 | 0·08§ | 89/116 | 27·7 | <0·001 | 40·4 | <0·001 |
| | | 5 | | | 52/52 | 14·4 | | 30·1 | |
| II | 3 | 9 | 1 | 0·08 | 51/66 | 23·0 | <0·01 | 37·8 | <0·01 |
| | | 3 | | | 32/32 | 14·0 | | 29·9 | |
| III | 3 | 7 | 2 or 4 | 0·03 to | 48/58 | 24·1 | <0·001 | 37·9 | <0·001 |
| | | 3 | | 0·06§§ | 29/30 | 15·3 | | 29·5 | |
| IV | 2 | 4 | 1 | 0·06 | 18/25 | 23·8 | <0·05 | 38·7 | <0·05 |
| | | 2 | | | 20/20 | 15·7 | | 31·8 | |
| V | 3 | 5 | 1 | 0·04 | 27/33 | 23·0 | <0·02 | 38·2 | <0·02 |
| | | 3 | | | 29/30 | 15·8 | | 30·5 | |
| VI | 5 | 7 | 1 | 0·02 | 56/57 | 15·2 | NS | 31·0 | NS |
| | | 5 | | | 49/50 | 15·5 | | 30·4 | |
| I | 5 | 9 | 2 or 4 | 0·08 | 89/116 | 27·7 | <0·05 | 40·4 | NS |
| II | 3 | 9 | 1 | 0·08 | 51/66 | 23·0 | | 27·8 | |

* 5–20 mice per group.

** All injections were given s.c.; cells treated with 0·02% GA were used for last immunization; immunization: $5 \times 10^5$ to $5 \times 10^6$ cells per mouse; challenge: $10^4$ viable tumour cells.

† MTD—mean time to tumour detection.

†† All tumour-bearing animals died during observation; MTM—mean time of mortality.

§ Cells treated with 0·06% GA and 0·04% GA were used subsequently in 6 groups receiving intermediate injections.

§§ Cells treated with 0·03% GA were used for the intermediate injection in groups receiving 3 injections (2 groups); intermediate injections of 0·04% GA-treated and 0·03% GA-treated cells were given in groups receiving 4 injections (2 groups).

††† *P*—degree of significance was calculated by Student's *t* test; significant protection was obtained in 8/9 individual groups of Pool I, 5/9 of Pool II, 4/7 Pool III, 2/4 of Pool IV, 1/5 of Pool V and 0/7 of Pool VI.

also been claimed that modification of cells by GA reduced the antibody response, whereas the cellular response was maintained and even increased (Dennert & Tucker, 1972; Parish, 1972). Preferential elicitation of cell-mediated immunity was assumed to be advantageous for induction of specific protective immunization against tumours (Mitchison, 1970).

The present study was undertaken to investigate the possibility of induction of antitumour protection against murine myeloma MOPC-315 in syngeneic BALB/c mice by use of GA-treated cells. The MOPC-315 myeloma cell line is derived from primary plasmacytoma tumour induced in mice of the BALB/c strain by i.p. administration of Bayol F, develops exclusively in this strain and is characterized by its ability to secrete anti-TNP IgA $\lambda_2$ immunoglobulin (Eisen *et al.*, 1968). This tumour cell line is highly tumorigenic by either i.p. or s.c. injection, and has been reported as weakly immunogenic (Williams & Kruger, 1972).

Suspensions of MOPC-315 cells prepared from s.c. induced tumours were used in all the experiments. The treatment with GA was performed by mixing volumes of 0·2 ml of cell suspensions ($2 \times 10^7$ viable cells/ml) in PBS at pH 7·2 with 1·8 ml GA solution (TAAB, England), for 10 min at room temperature. The treated cells were washed by 3 subsequent centrifugations at 100 *g* for 7 min at 4°C. Packed cells were resuspended either in Eagle's medium for thymidine (dT) incorporation measurements (Diamantstein & Ulmer, 1975) or in PBS for injections. The results in Fig. 1A show that treatment of cells by a solution of 0·00125% GA led to a marked decrease in (dT) incorporation whilst GA concentrations of 0·01–0·08% almost completely inhibited the incorporation. As shown in the same figure, the viability of cells, as measured by trypan-blue dye exclusion, was less affected by GA; marked reduction was obtained only at a concentration of at least 0·06% GA. MOPC-315 cells treated with less than 0·01% GA retained their ability to induce tumours. Partial loss of this ability (delay in development of tumours and reduction in mortality) was seen after treatment with 0·01–0·02% GA (Fig. 1B). Complete loss of the capacity to induce tumour was achieved by treatment with 0·06–0·08% GA (results not shown here). In this particular case, the cell suspension used was the same as in the experiment illustrated in Fig. 1A.

FIG. 1.—Effect of treatment with various concentrations of GA on MOPC-315 cells originated from s.c. induced tumours; $4 \times 10^6$ cells/0·2 ml were mixed with 1·8 ml of GA solution and incubated for 10 min at room temperature. A—GA-treated cells subcultured for 24 h; [$^3$H]-dT added 6 h before the end of incubation time. □—% of [$^3$H]-dT incorporation (levels in untreated cultures (2797 ± 496) taken as 100%). ○—% viability (levels in untreated cultures taken as 100%). B—Tumorigenicity of GA-treated cells; $5 \times 10^5$ cells per mouse s.c.; 8–10 mice per group. GA concentration (%): - - - 0·02; + + + 0·01; ·–·–· 0·005; —— none.

FIG. 2.—Protective effect of s.c. injection (21 and 7 days before challenge) with GA-treated MOPC-315 cells ($5 \times 10^5$/injection) originated from s.c. tumour; Challenge of $10^4$ viable cells given s.c. 7 days after the last immunizing injection. GA concentrations varied (as indicated) between 0·02 and 0·08%.

*Immunization schedule**

——★——★——★—— 0·08(28)–0·02(7)–challenge
– –▲– – –▲– – –▲– – 0·06(28)–0·02(7)–challenge
– –△– – –△– – –△– – 0·04(28)–0·02(7)–challenge
—+— —+— —+— 0·02(7)–challenge
———————— untreated–challenge

* The death rate in the group immunized by one injection of 0·02% GA-treated cells was significantly higher ($P < 0{\cdot}001$) than in the control unimmunized group.

Induction of antitumour protection by immunization with MOPC-315 GA-treated cells was investigated under various conditions, including GA concentrations used for treatment, number of immunizing injections, number of cells per injection, interval between injections and number of days between the last immunizing injection and challenge. The results summarized in the Table demonstrate that immunization with GA-treated MOPC-315 cells induced partial, though significant, protection against challenge with $10^4$ tumour cells. The antitumour protection was more marked in groups receiving more than one immunizing injection, especially if the first injection was with cells treated with 0·08% GA. A typical experiment is illustrated in Fig. 2.

The results show that the effect of GA was directly proportional to its concentration: firstly, impairment of dT incorporation, secondly, loss of tumorigenic ability and finally, change in trypan-blue dye exclusion. Correlation between the concentration of GA and the effect on tumour cells has also been reported by other workers (Bubbers & Henney, 1975; Kataoka *et al.*, 1975; Price *et al.*, 1979). Immunization with MOPC-315 cells treated with GA afforded partial protection against tumorigenic challenge. It would be of interest to devise more optimal schedules for immunization, and to clarify the nature of the immune response in protected and unprotected animals submitted to a similar schedule of immunization.

The authors would like to thank Mrs A. Jackman for her excellent technical assistance and Professor D. Givol of the Weizmann Institute of Science, Rehovot, Israel, for kindly providing us with the line of BALB/c myeloma MOPC-315 cells. The work was supported in part by a donation from Mrs Zelma Obrart, London, in memory of her late husband.

### REFERENCES

BUBBERS, J. E. & HENNEY, C. S. (1975) Studies on the synthetic capacity and antigenic expression of glutaraldehyde-fixed target cells. *J. Immunol.*, **114**, 1126.

CSABA, G. (1967) Attempts to induce antitumour immunity with living attenuated cells. *Neoplasma*, **14**, 167.

DENNERT, G. & TUCKER, D. F. (1972) Selective priming of T cells by chemically altered cell antigens. *J. Exp. Med.*, **136**, 656.

DIAMANTSTEIN, T. & ULMER, A. (1975) The antagonistic action of cyclic GMP and cyclic AMP on proliferation of B and T lymphocytes. *Immunology*, **28**, 113.

EISEN, H. N., SIMMS, E. S. & POTTER, M. (1968) Mouse myeloma proteins with anti hapten antibody activity. The protein produced by plasma cell tumor MOPC-315. *Biochemistry*, **7**, 4126.

FROST, P. & SANDERSON. C. J. (1975) Tumor immunoprophylaxis in mice using glutaraldehyde-treated syngeneic tumor cells. *Cancer Res.*, **35**, 2646.

FROST, P., EDWARDS, A. & SANDERSON, C. (1976) The use of glutaraldehyde fixation for the study of the immune response to syngeneic tumor antigen. *Ann. N.Y. Acad. Sci.*, **276**, 91.

KATAOKA, T., TSUKAGOSHI, S. & SAKURAI, Y. (1975) Transplantability of L1210 cells in BALB/c x $DBA2F_1$ mice associated with cell agglutinability by concanavalin A. *Cancer Res.*, **35**, 531.

KATAOKA, T., OH-HASHI, F., TSUKAGOSHI, S. & SAKURAI, Y. (1977*a*) Induction of resistance to L1210 leukemia in BALB/c x $DBA2CrF_1$ mice, with L1210 cells treated with glutaraldehyde and concanavalin A. *Cancer Res.*, **37**, 964.

KATAOKA, T., OH-HASHI, F., TSUKAGOSHI, E. & SAKURAI, Y. (1977*b*) Enhanced induction of immune resistance by concanavalin A-bound L1210 vaccine and an immunopotentiator prepared from *Coriolus versicolor*. *Cancer Res.*, **37**, 4416.

KATAOKA, T., KOBAYASHI, H. & SAKURAI, Y. (1978) Potentiation of concanavalin A-bound L1210 vaccine *in vivo* by chemotherapeutic agents. *Cancer Res.*, **38**, 1202.

KATAOKA, T., OH-HASHI, F. & SAKURAI, Y. (1979*a*) Blastogenic potency of concanavalin A-bound L1210 leukemia vaccine associated with its immunogenic activity. *Gann*, **70**, 155.

KATAOKA, T., OH-HASHI, F., SAKURAI, Y., OKABE, M. & GOMI, K. (1979*b*) Factors responsible for immune resistance to L1210 murine leukemia in hyperimmune mice. *Cancer Immunol. Immunother.*, **7**, 123.

KATAOKA, T., TSUKAGOSHI, S., SAKURAI, Y. & OKABE, M. (1979*c*) Potentiation of L1210 murine leukemia vaccine *in vivo* by levamisole. *Gann*, **70**, 515.

KATAOKA, T., OH-HASHI, F. & SAKURAI, Y. (1979*d*) Immunotherapeutic response of concanavalin A-bound L1210 vaccine enhanced by a streptococcal immunopotentiator, OK-432. *Cancer Res.*, **39**, 2807.

KLUCHAREVA, T. E., MATVEEVA, V. A. & DEICHMAN, G. I. (1978) Sensitivity of TSTA and species-specific cell membrane antigens of tumor cells to glutaraldehyde treatment. *Neoplasma*, **25**, 273.

MITCHISON, N. A. (1970) Immunologic approach to cancer. *Transplant Proc.*, **2**, 92.

PARISH, C. R. (1972) Preferential induction of cell mediated immunity by chemically modified sheep erythrocytes. *Eur. J. Immunol.*, **2**, 143.

POWELL, P. C. (1975) Immunity to Marek's disease induced by glutaraldehyde-treated cells of Marek's disease lymphoblastoid cell lines. *Nature*, **257**, 684.

PRICE, M. R., DENNICK, R. G., ROBINS, R. A. & BALDWIN, R. W. (1979) Modification of the immunogenicity and antigenicity of rat hepatoma cells. I. Cell-surface stabilization with glutaraldehyde. *Br. J. Cancer*, **39**, 621.

RICHARDS, F. M. & KNOWLES, J. R. (1968) Glutaraldehyde as a protein cross linking reagent. *J. Mol. Biol.*, **37**, 231.

SANDERSON, C. J. & FROST, P. (1974) The induction of tumour immunity in mice using glutaraldehyde-treated tumour cells. *Nature*, **248**, 690.

TOMECKI, J. (1979) The influence of immunization of Syrian hamsters with tumor cells treated with glutaraldehyde on transplantation immunity and the cytotoxic effect of lymphocytes on polyoma tumor cells. *Arch. Immunol. Therap. Exp.*, **27**, 209.

WILLIAMS, W. H. & KRUGER, R. G. (1972) Tumor-associated transplantation antigens of myelomas induced in BALB/c mice. *J. Nat. Cancer Inst.*, **49**, 1613.


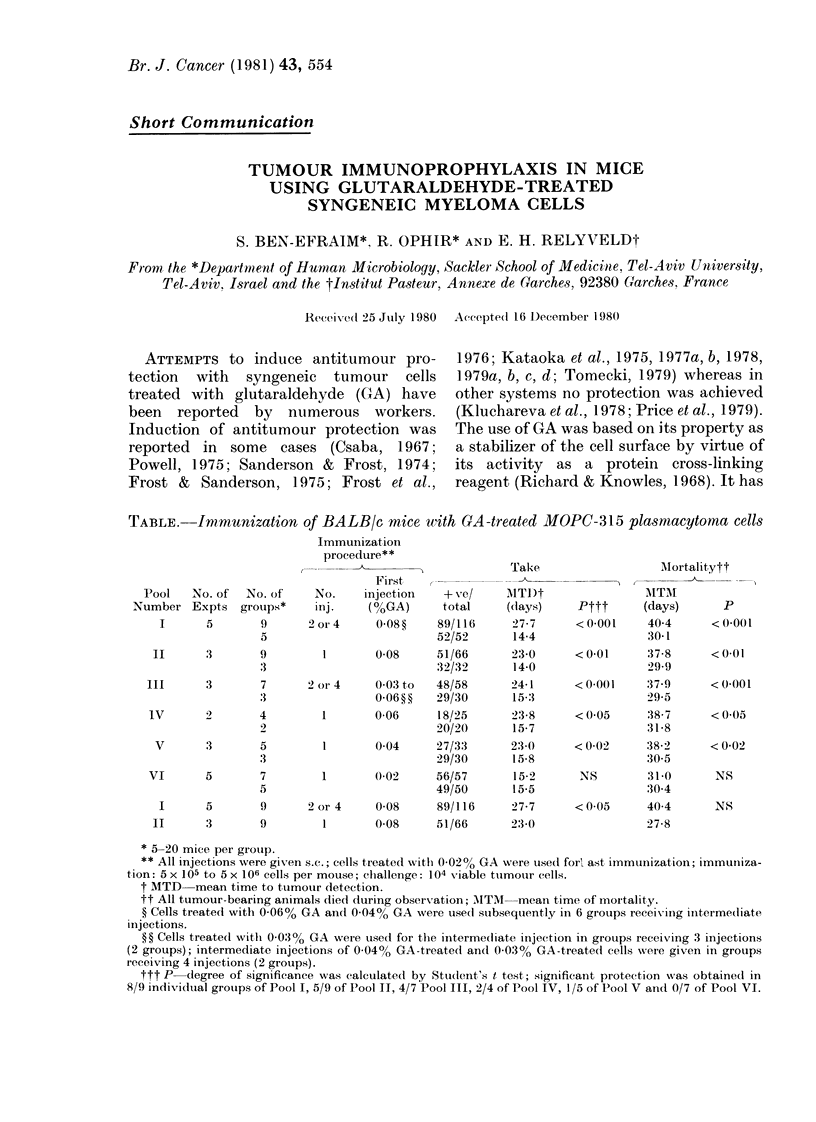

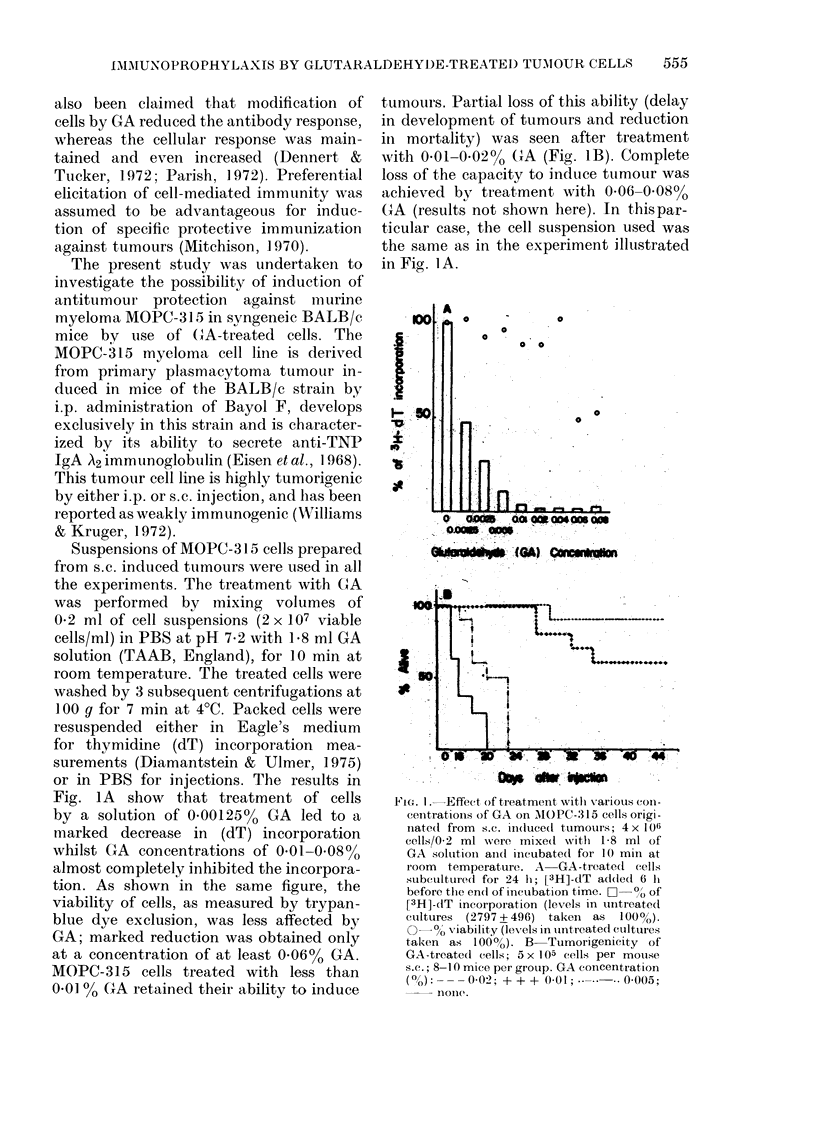

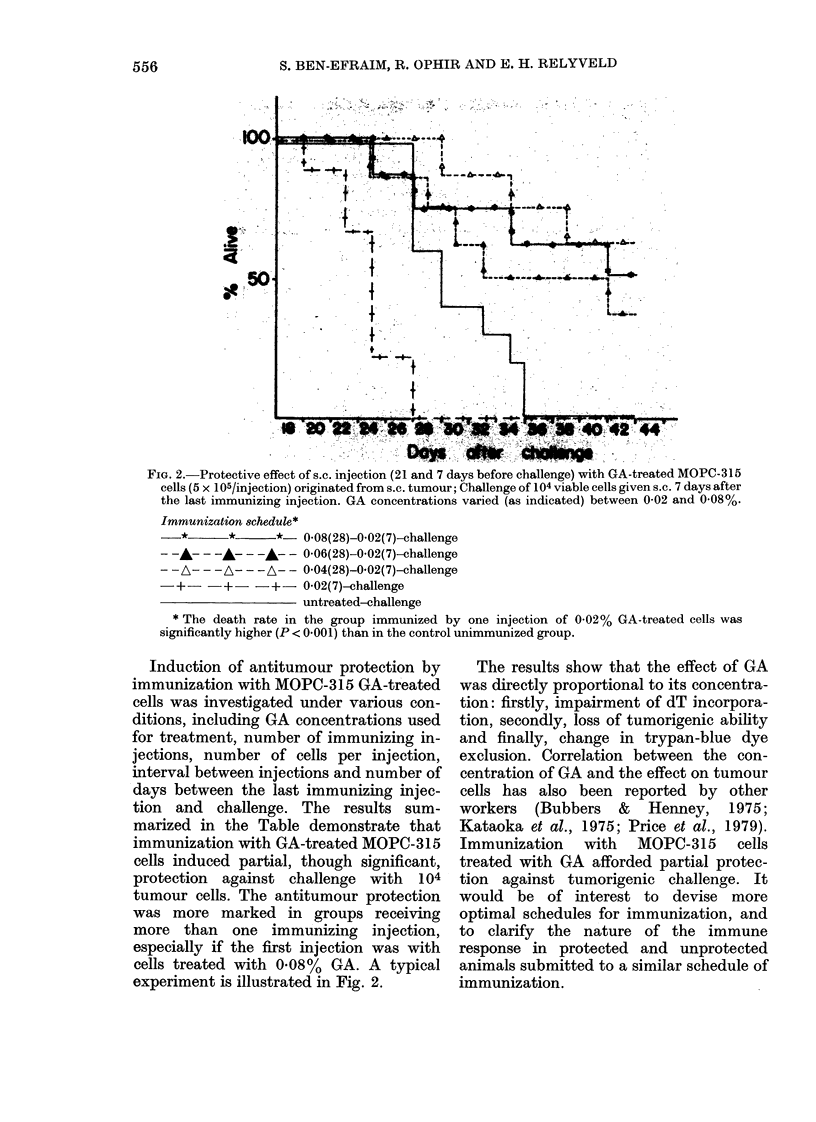

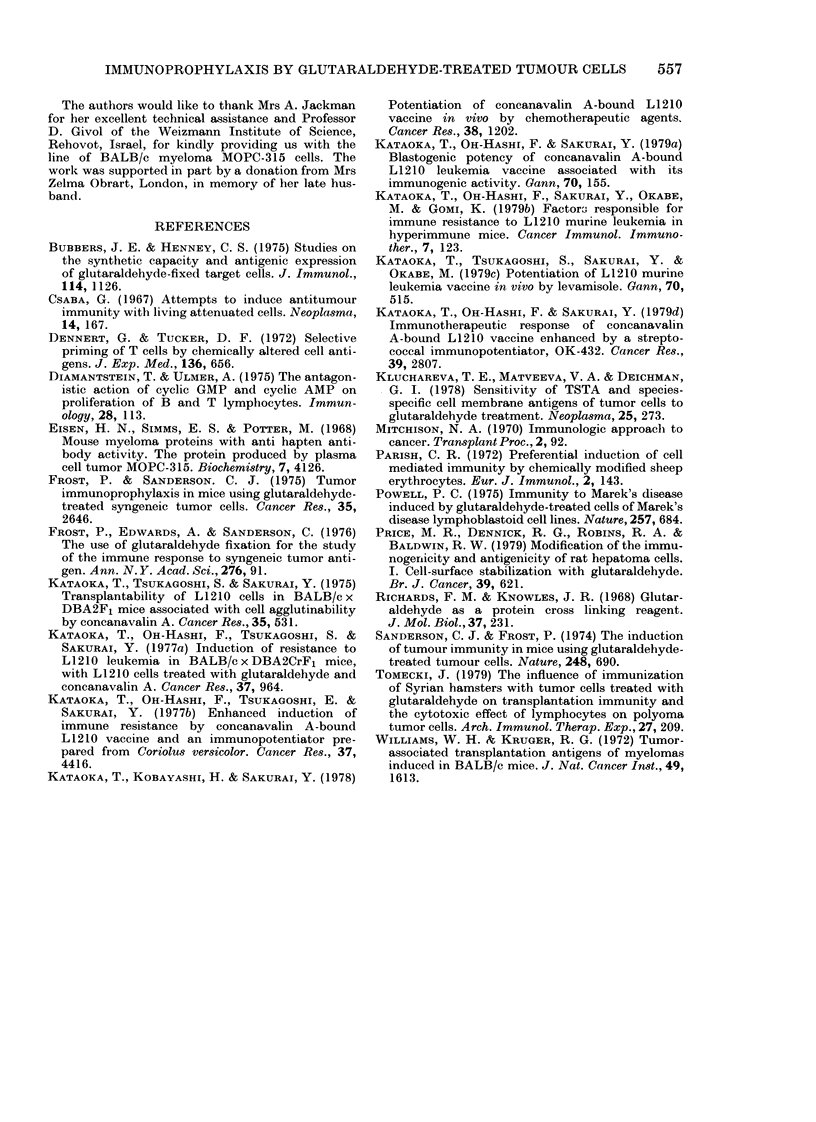


## References

[OCR_00356] Bubbers J. E., Henney C. S. (1975). Studies on the synthetic capacity and antigenic expression of glutaraldehyde-fixed target cells.. J Immunol.

[OCR_00362] Csaba G. (1967). Attempts to induce antitumour immunity with living attenuated cells.. Neoplasma.

[OCR_00367] Dennert G., Tucker D. F. (1972). Selective priming of T cells by chemically altered cell antigens.. J Exp Med.

[OCR_00372] Diamantstein T., Ulmer A. (1975). The antagonistic action of cyclic GMP and cyclic AMP on proliferation of B and T lymphocytes.. Immunology.

[OCR_00378] Eisen H. N., Simms E. S., Potter M. (1968). Mouse myeloma proteins with antihapten antibody acitivity. The protein produced by plasma cell tumor MOPC-315.. Biochemistry.

[OCR_00390] Frost P., Edwards A., Sanderson C. (1976). Discussion paper: the use of glutaraldehyde fixation for the study of the immune response to syngeneic tumor antigen.. Ann N Y Acad Sci.

[OCR_00384] Frost P., Sanderson C. J. (1975). Tumor immunoprophylaxis in mice using glutaraldehyde-treated syngeneic tumor cells.. Cancer Res.

[OCR_00417] Kataoka T., Kobayashi H., Sakurai Y. (1978). Potentiation of concanavalin A-bound L1210 vaccine in vivo by chemotherapeutic agents.. Cancer Res.

[OCR_00423] Kataoka T., Oh-hashi F., Sakurai Y. (1979). Blastogenic potency of concanavalin-A-bound L1210 leukemic vaccine associated with its immunogenic activity.. Gan.

[OCR_00409] Kataoka T., Oh-hashi F., Tsukagoshi S., Sakurai Y. (1977). Enhanced induction of immune resistance by concanavalin A-bound L1210 vaccine and an immunopotentiator prepared from Coriolus versicolor.. Cancer Res.

[OCR_00402] Kataoka T., Oh-hashi F., Tsukagoshi S., Sakurai Y. (1977). Induction of resistance to L1210 leukemia in BALB/c X DBA/2Cr F1 mice, with L1210 cells treated with glutaraldehyde and concanavalin A.. Cancer Res.

[OCR_00436] Kataoka T., Tsukagoshi S., Sakurai Y., Okabe M. (1979). Potentiation of L1210 murine leukemia vaccine in vivo by levamisole.. Gan.

[OCR_00449] Kluchareva T. E., Matveeva V. A., Deichman G. I. (1978). Sensitivity of TSTA and species-specific cell membrane antigens of tumor cells to glutaraldehyde treatment.. Neoplasma.

[OCR_00455] Mitchison N. A. (1970). Immunologic approach to cancer.. Transplant Proc.

[OCR_00459] Parish C. R. (1972). Preferential induction of cell-mediated immunity by chemically modified sheep erythrocytes.. Eur J Immunol.

[OCR_00464] Powell P. C. (1975). Immunity to Marek's disease induced by glutaraldehyde-treated cells of Marek's disease lymphoblastoid cell lines.. Nature.

[OCR_00468] Price M. R., Dennick R. G., Robins R. A., Baldwin R. W. (1979). Modification of the immunogenicity and antigenicity of rat hepatoma cells. I. Cell-surface stabilization with glutaraldehyde.. Br J Cancer.

[OCR_00480] Sanderson C. J., Frost P. (1974). The induction of tumour immunity in mice using glutaraldehyde-treated tumor cells.. Nature.

[OCR_00485] Tomecki J. (1979). The influence of immunization of Syrian hamsters with tumor cells treated with glutaraldehyde on transplantation immunity and the cytotoxic effect of lymphocytes on polyoma tumor cells.. Arch Immunol Ther Exp (Warsz).

[OCR_00491] Williams W. H., Krueger R. G. (1972). Tumor-associated transplantation antigens of myelomas induced in BALB-c mice.. J Natl Cancer Inst.

